# Plasma‐based microRNA signatures in early diagnosis of breast cancer

**DOI:** 10.1002/mgg3.1092

**Published:** 2020-03-02

**Authors:** Xu Li, Wenjing Zou, Yuzhen Wang, Zijun Liao, Lina Li, Yang Zhai, Lingxiao Zhang, Shanzhi Gu, Xinhan Zhao

**Affiliations:** ^1^ Department of Medicine Oncology Affiliated Hospital of Medical College of Xi'an Jiaotong University (Shaanxi Provincial Cancer Hospital) Xi'an China; ^2^ Department of Geriatrics Xi'an No 5 Hospital Xi'an China; ^3^ Department of Medicine Oncology The First Affiliated Hospital of Xi'an Jiaotong University Xi'an China; ^4^ College of Forensic Medicine Xi'an Jiaotong University Health Science Center Xi'an China

**Keywords:** biomarker, breast cancer, miRNAs, plasma

## Abstract

**Background:**

MicroRNAs (miRNAs) play an important role in the development and progression of breast cancer (BC). The purpose of the present study was to identify plasma miRNAs enabling early diagnosis of BC.

**Materials and Methods:**

Expression levels of seven plasma miRNAs (miR‐23a‐3p, miR‐29b‐2‐5p, miR‐130a‐5p, miR‐144‐3p, miR‐148a‐3p, miR‐152‐3p, and miR‐182‐5p) in 106 patients with newly diagnosed BC and 96 healthy participants were analyzed by qRT‐PCR. We also evaluated the relationship between the expression levels of these miRNAs and clinicopathological features of patients with BC.

**Results:**

Compared with healthy controls, we found that miR‐23a‐3p (*p* = .025), miR‐130a‐5p (*p* = .006), miR‐144‐3p (*p* = .040), miR‐148a‐3p (*p* = .023), and miR‐152‐3p (*p* = .019) were downregulated in the plasma of patients with BC. MiR‐130a‐5p, miR‐144‐3p, and miR‐152‐3p were downexpressed in BC tissues as well as plasma. The expression of the miR‐23a‐3p, miR‐144‐3p, and miR‐152‐3p was related to ER positive and PR positive. Besides, miR‐23a‐3p, miR‐144‐3p, and miR‐152‐3p did show the significant difference in the staging compromised to the control, especially in stage I‐II. Moreover, we also found that miR‐144‐3p and miR‐148a‐3p were associated with lymph node invasion.

**Conclusions:**

The expression levels of the miR‐23a‐3p, miR‐130a‐5p, miR‐144‐3p, miR‐148a‐3p, and miR‐152‐3p were lower in patients with BC compared to healthy controls and were associated with ex hormone receptor, clinical stage, and lymph node metastasis, indicating the diagnostic potential of these miRNAs in BC.

## Graphical abstract

We identified significant reduction of miR‐23a‐3p, miR‐130a‐5p, miR‐144‐3p, miR‐148a‐3p, and miR‐152‐3p in the plasma of the patients compared with the controls. MiR‐130a‐5p, miR‐144‐3p, and miR‐152‐3p were downexpressed in BC tissues as well as plasma in silico analysis. The relative expression of these five miRNAs was closely associated with the clinicopathologic features of the BC such as the expression of ER, PR, and HER2, histological tumor grades, and lymph node metastasis.

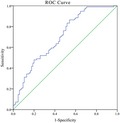

## 1 INTRODUCTION

Breast cancer (BC) is the most commonly diagnosed cancer and the leading cause of cancer death among females (Bray et al., 2018). In China, there were about 268,600 newly diagnosed female BC cases, accounting for 15% of all new cancers in women, and 69,500 deaths in 2015(Chen et al., 2016). Although the mortality rates have continued to decrease over the years due to the advances made in early diagnosis and treatment, tens of thousands of women die from this disease each year. Early detection of BC is one of the main prerequisites for successful treatment and reduction in mortality from this disease (Coleman, 2017). Moreover, it also plays a critical role in the diagnosis of BC, monitoring of the disease progression and response to treatment (Nattinger & Mitchell, 2016). Despite the fact that mammography, ultrasonography, and magnetic resonance imaging diagnosis for BC are the currently used noninvasive screening tool, there are some concerns about the high rates of misdiagnosis, missed diagnoses, and overdiagnosis of this malignancy (Bleyer & Welch, 2012). Thus, there is a need for the development of novel noninvasive biomarkers to improve the early diagnosis of malignant breast lesions. Available evidence suggests that serum/plasma miRNA levels may be used as noninvasive biomarkers for early BC diagnosis (Hamam et al., 2017).

MicroRNAs (miRNAs) are a class of small, approximately 19–25 nucleotides of noncoding RNAs that regulate gene expression at post‐transcriptional level (Bartel, 2004). Evidence suggests that miRNA is associated with the development and progression in a number of human diseases, including cancer (Cai et al., 2017; Ding et al., 2017; Li et al., 2013). MiRNAs are involved in multiple biological processes of BC, such as differentiation, proliferation, apoptosis, development, and tumorigenesis (Yahya & Elsayed, 2015). MiRNA expression may be used as potential biomarkers for the diagnosis, prognosis, and therapy. Nevertheless, most of the studies describe the miRNA expression in cell lines and primary tumor tissue to date. Recently, several reports have proved that circulating miRNAs derived from epithelial tumors were deregulated in different types of diseases, including cancer (Cheng, 2015). The distinct biological properties of the serum/plasma miRNAs including the remarkable stability, availability for rapid and accurate quantification, the possibility of their repeated measurement, and a direct link with disease states make them attractive candidates for the development of novel markers (McDonald, Milosevic, Reddi, Grebe, & Algeciras‐Schimnich, 2011).

Recently, several reports have shown the dysregulated expression of miR‐23a‐3p, miR‐29b‐2‐5p, miR‐130a‐5p, miR‐144‐3p, miR‐148a‐3p, miR‐152‐3p, and miR‐182‐5p in tumor tissue was associated with the development of BC (Chen, Cheng, Chen, Chen, & Shen, 2017; Gu et al., 2018; Kong, Zhang, Li, Shao, & Fang, 2018). However, there have been a few reports on the role of plasma miRNAs in BC. Here, we investigated and compared the expression of these seven plasma miRNAs in patients with BC and healthy controls. Moreover, potential relationships between these plasma miRNAs level and existing clinicopathological features of BC, such as hormone receptor status, stage, clinical T, and lymph node invasion, were statistically analyzed. Our data showed that miR‐23a‐3p, miR‐130a‐5p, miR‐144‐3p, miR‐148a‐3p, and miR‐152‐3p were significantly downregulated in plasma of patient with BC and could be served as the potential noninvasive molecular biomarkers for the early detection of BC.

## 2 SUBJECTS AND METHODS

### 2.1 Study subjects and sample collection

The study consisted 106 patients with BC and 96 healthy age‐matched volunteers from the First Affiliated Hospital of Xi'an Jiaotong University. All participants were genetically unrelated ethnic Han Chinese females. Patients with BC were newly diagnosed and histopathologically confirmed by biopsy and pathology. No patients had undergone chemotherapy or radiotherapy, surgical therapy, or systemic therapy before sample collection. The healthy controls had no history of cancer, no family history of BC, and no endocrine or reproductive system diseases by physical examination. Clinical characteristics information was collected through interviewer‐administered questionnaires and/or medical records.

Five milliliters of blood sample from each participant was collected into EDTA tube for miRNA analysis. Plasma was subsequently isolated by centrifugation at 3,000 rpm for 10 min at 4°C for the isolation of RNA. This study was approved by the institutional ethics committees of the First Affiliated Hospital of Xi'an Jiaotong University. All procedures performed in studies involving human participants were in accordance with the 1964 Helsinki Declaration. Written informed consent was obtained from all individuals who participated in the study.

### 2.2 Plasma RNA extraction and miRNA qRT‐PCR

Total RNA (including miRNAs) was extracted from plasma using the miRcute Serum/plasma miRNA isolation kit (cat. no. DP501; Tiangen) following the manufacturer's instructions. After washing miRNAs were eluted in 30 μl of RNase‐free ddH_2_O. Quality of RNA was assessed by NanoDrop 2000C (Thermo Scientific). Chromatographic characteristics and integrity of all RNA samples were determined by RNA 6000 Nano LabChip (Agilent Technologies) and Agilent 2100 Bioanalyzer system (Agilent Technologies). Then, RNA samples were converted into cDNA immediately.

The reverse transcription (RT) reactions of U6 and miRNAs (miR‐23a‐3p, miR‐29b‐2‐5p, miR‐130a‐5p miR‐144‐3p, miR‐148a‐3p, miR‐152‐3p, and miR‐182‐5p) were performed by the miRcute Plus miRNA First‐Strand cDNA Synthesis Kit (cat. no. KR211; Tiangen) with Oligo (dT)‐Universal Tag primer. The synthesis reaction consisted of 10 μl 2 × miRNA RT reaction buffer, 2 μl miRNA RT enzyme Mix, 0.6–2 μg total RNA, and RNase‐free ddH_2_O up to 20 μl. The synthesis reaction conditions were 42°C for 60 min and 95°C for 3 min, and hold at 4°C. After the reaction, the product was diluted with 200 μl RNase‐free water and kept at −20°C until further use.

SYBR Green‐based qPCR profiling was performed using miRcute Plus miRNA qPCR Detection Kit (cat. no. FP411; Tiangen) in ABI 7500 Real‐Time PCR System (Applied Biosystems) according to the manufacturer's instructions. The miRNA‐specific forward primers sequences were designed based on the miRNA sequences obtained from the miRBase database (Table S1). The amplification reaction system consisted of 10 μl 2 × miRcute plus miRNA premix (with SYBR&ROX), 0.4 μl forward primer (10 μM), 0.4 μl universal reverse primer (10 μM), 2 μl cDNA, and RNase‐free ddH_2_O up to 20 μl. The reaction was carried out at 95°C for 15 min, followed by 40 cycles at 94°C for 20 s and 60°C for 34 s, and hold at 4°C. Triplicate wells were measured for each sample. RT reactions and SYBR Green Real‐Time PCR were carried out in a blinded manner. Appropriate negative controls (ddH_2_O or no template control) were used in both cDNA synthesis and RT‐qPCR reactions. The levels of miRNAs were normalized using U6 as reference RNA. The relative expression quantity (RQ) of miRNA was calculated as RQ = 2^−ΔΔ^ *^Ct^*. ΔΔ*Ct* = mean value of the study group (*Ct*_miRNA_ − *Ct*_U6_) – mean value of the control group (*Ct*_miRNA_ − *Ct*_U6_).

### 2.3 Statistical analyses

All analyses were performed with SPSS 18.0 software (SPSS Institute). Baseline characteristics were presented as mean ± standard deviation (*SD*) for continuous data and as number (percentages) for categorical parameters. Independent samples *t* test was used to evaluate the age distribution. Plasma miRNA levels between multiple groups were compared using the independent samples *t* test. The Spearman correlation coefficient was used for correlation analysis of expression levels of these miRNAs. The median expression level of miRNAs was used as the cutoff point to divide patients with BC into low and high expression groups. A chi‐square test and one‐way ANOVA were used to assess the association between miRNA levels and clinicopathological characteristics between cases and controls. Diagnostic accuracy of candidate miRNAs or their combinations was assessed by receiver operating characteristic (ROC) curves analysis, and the area under the ROC curve (AUC) was also calculated. All tests were two‐sided test and *p* values less than .05 was considered to be statistically significant. Statistical significance was established at *p < .05 or **p < .01.

### 2.4 TCGA data

Due to the purpose of relatively early diagnosis in this study and plasma collected precancer therapy, tumor tissue was not available from patients recruited for this study. In addition, plasma collected from all patients enrolled in this study occurred prior to chemotherapy administration and/or tumor biopsy. Therefore, in order to compare differences in miRNA expression levels in tumor tissue versus plasma, publicly available data from the TCGA (Cancer Genome Atlas Network, 2012) were used. The miRNA expression data of the cancer tissues from patients with BC were downloaded from TCGA (https://portal.gdc.cancer.gov/) in the form of data matrices documenting patterns of miRNA expression for 870 BC tissue samples and 78 normal tissues at age 20–75 years. The tissue level expression of miRNA (miRNA‐seq), available as RPKM (reads per kilobase of transcript per million) values, was obtained using the Illumina HiSeq platform. The expression level of miRNA was normalized using Limma package of R language (Smyth, 2004).Then, we analyzed the expression trends of a given miRNA across tissue and plasma samples. In addition, the miRNA expression and prognostic significance in BC were evaluated using Kaplan‐Meier Plotter database (http://kmplot.com/analysis/index.php?p=service%26cancer=breast_mirna).

## 3 RESULTS

### 3.1 Characteristics of study subjects

A total of 202 women which included 106 patients (median age 53.19 ± 6.41 years) with BC and 96 healthy controls (median age 54.81 ± 8.38 years) were enrolled in the study. There were no significant differences in age between the patients with BC and the controls (*p* = .122). Characteristics of study subjects were summarized in Table 1, including age, hormone‐receptor‐positive status, HER2 status, stage, T classification, nodal status, and pathological subtypes.

**Table 1:** Characteristics of patients with breast cancer and controls

Variables		Case (*n* = 106)		Control (*n* = 96)		*p*
		Number	%	Number	%	
Age	Mean ± *SD* (year)	53.19 ± 6.41		54.81 ± 8.38		**.122**
ER status	Positive	66	62.3			
	Negative	31	29.2			
	Missing	9	8.5			
PR status	Positive	57	53.8			
	Negative	39	36.8			
	Missing	10	9.4			
HER2 status	Positive	30	28.3			
	Negative	49	46.2			
	Missing	27	25.5			
Stage	I‐II	63	59.4			
	III‐IV	38	35.8			
	Missing	5	4.7			
Clinical T	1–2	86	81.1			
	3–4	14	13.2			
	Missing	6	5.6			
Lymph node invasion	Yes	55	51.9			
	No	46	43.4			
	Missing	5	4.7			
Pathological subtypes	Luminal B	52	47.7			
	Luminal A	23	21.1			
	Her‐2	19	17.4			
	Triple negative	10	9.2			
	Missing	5	4.6			

Note

Bold *p* values were calculated by independent samples T test.

Abbreviations: ER, estrogen receptor; HER2, human epidermal growth factor receptor 2; RP, progesterone receptor.

### 3.2 Significantly differentiated plasma miRNAs between the controls and BC patients

**Figure 1:**
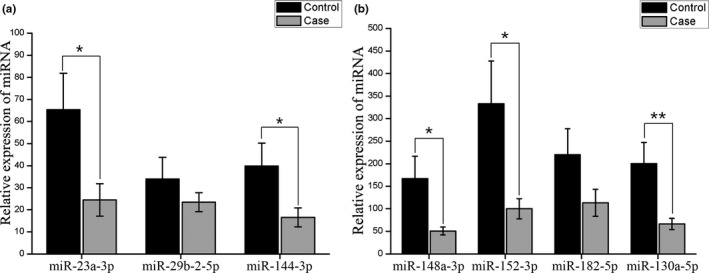
The expression of seven plasma miRNAs in breast cancer patients and healthy controls. Statistical significance of expression level with * for *p* < .05 and ** for *p* < .01

We measured plasma miRNA levels of miR‐23a‐3p, miR‐29b‐2‐5p, miR‐130a‐5p, miR‐144‐3p, miR‐148a‐3p, miR‐152‐3p, and miR‐182‐5p between the controls and patients with BC. We found that miR‐23a‐3p (*p* = .025), miR‐130a‐5p (*p* = .006), miR‐144‐3p (*p* = .040), miR‐148a‐3p (*p* = .023), and miR‐152‐3p (*p* = .019) were downregulated in the plasma of patients with BC when compared with healthy women (Table 2 and Figure 1).

**Table 2:** Expression changes of plasma miRNA in BC cases and healthy controls

miRNAs	Plasma			Tissue (TCGA)		
	Controls (*M* ± *SD*)	BC patients (*M* ± *SD*)	*p*	Controls (*M* ± *SD*)	BC patients (*M* ± *SD*)	*p*
miR‐23a‐3p	65.37 ± 16.43	24.48 ± 7.35	**.025** *	3,545.35 ± 127.80	3,476.15 ± 77.20	.644
miR‐130a‐5p	200.62 ± 46.65	66.38 ± 12.32	**.006** **	134.48 ± 66.14	84.61 ± 21.77	**<.001** **
miR‐144‐3p	39.87 ± 10.36	16.54 ± 4.30	**.040** *	613.89 ± 154.24	70.48 ± 28.67	**.001** **
miR‐148a‐3p	167.03 ± 49.49	50.90 ± 8.83	**.023** *	30,384.03 ± 2,351.92	55,066.80 ± 1,650.86	**<.001** **
miR‐152‐3p	333.18 ± 94.81	100.13 ± 22.44	**.019** *	293.70 ± 112.36	261.84 ± 161.79	**.023** *

Abbreviations: BC, breast cancer; M, mean values; *SD*, standard deviation.

Bold indicates statistical significance of expression level with

a *p* < .05,

b *p* < .01.

Next, tissue expression trends in the corresponding five miRNAs were measured (Table 2). MiRNA expression values in BC tissue (*n* = 870) and noncancerous tissue (*n* = 78) were obtained from publically available TCGA RNASeq data. Expression trends for some miRNAs (miR‐130a‐5p, miR‐144‐3p and miR‐152‐3p) were similar in both tissue and plasma. The expression of miR‐23a‐3p and miR‐148a‐3p in tissue and plasma levels showed a reversal of the trend. MiR‐148a‐3p was a significantly increased in BC tissue levels (TCGA) compared with nontumor tissue samples, and miR‐23a‐3p showed no significantly different.

In addition, the correlation analyses showed that these plasma miRNA expression levels had strong correlations, and their correlation coefficients were 0.315 (miR‐23a‐3p vs. miR‐144‐3p, *p* = .001), 0.584 (miR‐23a‐3p vs. miR‐148a‐3p, *p* < .001), 0.289 (miR‐23a‐3p vs. miR‐152‐3p, *p* = .003), 0.412 (miR‐130a‐5p vs. miR‐148a‐3p, *p* < .001), 0.391 (miR‐144‐3p vs. miR‐148a‐3p, *p* < .001), 0.671 (miR‐144‐3p vs. miR‐152‐3p, *p* < .001), and 0.481 (miR‐148a‐3p vs. miR‐152‐3p, *p* < .001), respectively (Table 3).

**Table 3:** Coefficients of correlation among five miRNAs

miRNAs	miR‐23a‐3p		miR‐130a‐5p		miR‐144‐3p		miR‐148a‐3p		miR‐152‐3p	
	*r*	*p*	*r*	*p*	*r*	*p*	*r*	*p*	*r*	*p*
miR‐23a‐3p	1.000	—								
miR‐130a‐5p	0.146	.154	1.000	—						
miR‐144‐3p	0.315	**.001** **	0.060	.557	1.000	—				
miR‐148a‐3p	0.584	**<.001** **	0.412	**<.001** **	0.391	**<.001** **	1.000	—		
miR‐152‐3p	0.289	**.003** **	0.185	.068	0.671	**<.001** **	0.481	**<.001** **	1.000	—

Bold indicates statistical significance of expression level with

*p* < .05,

a *p* < .01.

### 3.3 The diagnosis role of candidate miRNAs for BC

**Figure 2:**
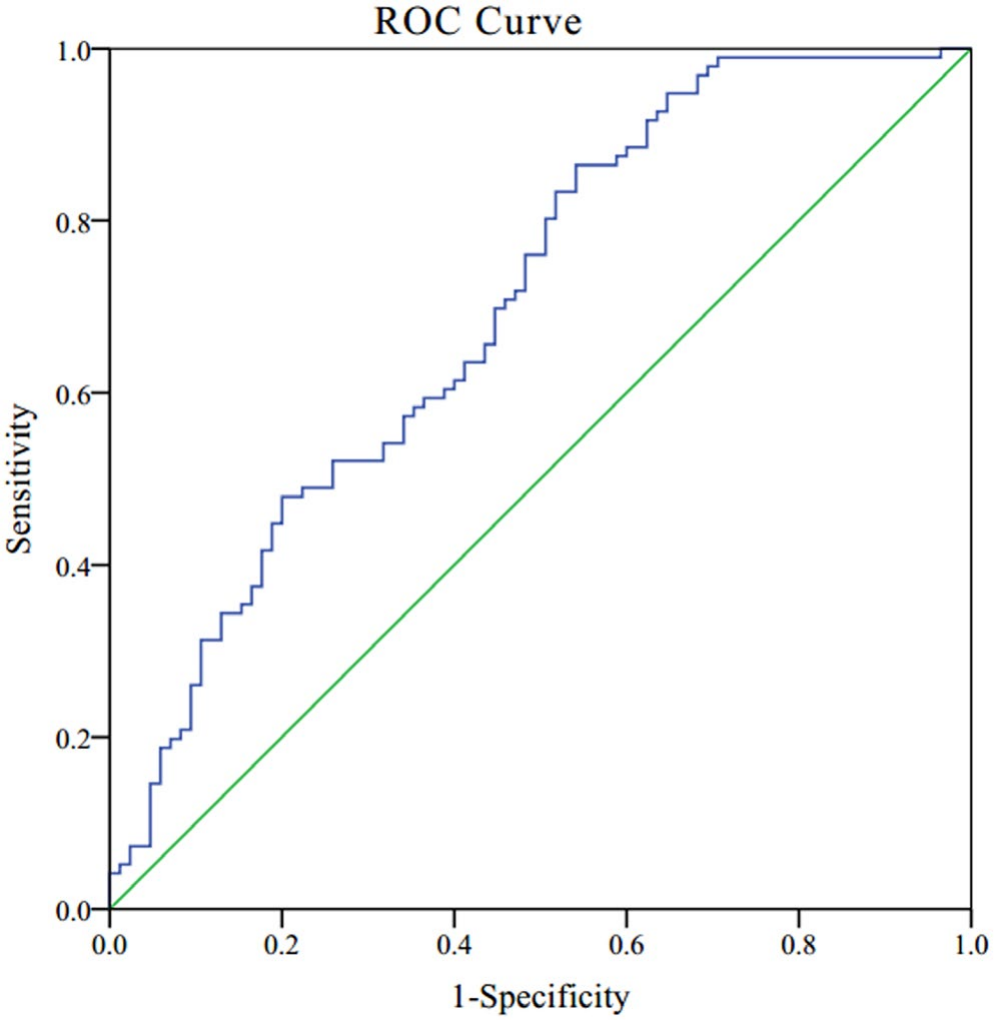
Receiver operating characteristics (ROC) curve of combined miRNA panel to differentiate patients with breast cancer from healthy controls

Receiver operating characteristics (ROC) curve was used to evaluate the ability of significantly differentiated plasma miRNAs to distinguish patients with BC from healthy controls (Figure 2). For combined plasma miRNA panel (miR‐23a‐3p, miR‐130a‐5p, miR‐144‐3p, miR‐148a‐3p, and miR‐152‐3p), the AUC was 0.699 with 95% CI: 0.622–0.775 and the sensitivity and specificity were 0.865 and 0.459, respectively.

### 3.4 Correlation of plasma miRNA levels to the status of ER, PR and HER2

Moreover, we have stratified the patients to examine the associations between the plasma levels of the designated miRNAs and the status of estrogen receptor (ER), progesterone receptor (PR), and human epidermal growth factor receptor 2 (HER2). We found that the miR‐23a‐3p (*p* = .037), miR‐130a‐5p (*p* = .007), miR‐144‐3p (*p* = .043), miR‐148a‐3p (*p* = .032), and miR‐152‐3p (*p* = .037) did show significant difference in ER‐positive patients compromised to the controls (Figure S1). MiR‐130a‐5p (*p* = .020), miR‐148a‐3p (*p* = .023), and miR‐152‐3p (*p* = .012) also showed significant differences between ER‐negative patients and the controls. As shown in Figure S2, the miR‐23a‐3p (*p* = .004), miR‐130a‐5p (*p* = .010), miR‐144‐3p (*p* = .006), miR‐148a‐3p (*p* = .035), and miR‐152‐3p (*p* = .010) were significantly downregulated in the patients with PR positive compared with the controls. Furthermore, miR‐130a‐5p (*p* = .011) and miR‐148a‐3p (*p* = .023) expressed statistically significantly different between two groups: PR‐negative patients and the controls. For HER2 status (Figure S3), we found that the miR‐23a‐3p (*p* = .002), miR‐130a‐5p (*p* = .007), miR‐144‐3p (*p* = .025), and miR‐152‐3p (*p* = .012) displayed significant differences in HER2‐negative patients with comparison to the healthy controls. The significant difference miR‐130a‐5p between HER2‐positive patients and the controls was observed (*p* = .044, Table 4).

**Table 4:** The correlation of relative levels of miRNAs with breast cancer patients' clinic pathological parameters

Characteristics	miR‐23a‐3p			miR‐130a‐5p			miR‐144‐3p			miR‐148a‐3p			miR‐152‐3p		
	Low	High	*p* value	Low	High	*p* value	Low	High	*p* value	Low	High	*p* value	Low	High	*p* value
Age (years)															
≤54	54	9	.481	46	14	.534	47	15	.577	46	17	.267	51	11	.271
>54	33	8		27	11		33	8		33	7		30	11	
ER status															
Positive	51	13	.377	48	13	.150	49	15	.734	50	14	.393	47	17	.291
Negative	27	4		18	10		22	8		21	9		25	5	
PR status															
Positive	44	11	.567	42	12	.292	43	12	.433	43	13	.677	42	13	.996
Negative	33	6		23	11		27	11		27	10		29	9	
HER2 status															
Positive	22	7	.127	15	11	**.044** *	20	8	.619	19	8	.555	22	6	.687
Negative	41	5		36	9		36	11		36	11		35	12	
Stage															
I‐II	50	13	.227	41	17	.320	48	14	.967	46	16	.691	48	13	.971
III‐IV	32	4		28	7		28	8		28	8		29	8	
Clinical T															
1–2	70	15	.814	59	20	.678	64	21	.171	65	19	.218	64	20	.189
3–4	11	2		2	4		12	1		8	5		12	1	
Lymph node invasion															
No	43	10	.631	36	13	.866	37	16	**.041** *	36	16	.124	39	13	.360
Yes	39	7		33	11		40	6		38	8		38	8	
Pathological subtypes															
Luminal A	20	2	.723	21	1	.683	19	3	.661	18	4	.996	20	1	.088
Luminal B	39	9		39	4		41	7		39	9		34	14	
Her‐2	16	3		16	3		15	3		15	3		16	2	
Triple negative	9	1		9	1		7	3		8	2		8	2	

Bold indicates statistical significance of expression level with

a *p* < .05.

### 3.5 Correlation of plasma miRNA levels to the status of pathological data

We also evaluated the correlation of these miRNAs and the pathological data, such as BC stage, T classification, nodal status, and pathological subtypes. Compared with normal controls, lower levels of miR‐23a‐3p (*p* = .025), miR‐130a‐5p (*p* = .021), miR‐144‐3p (*p* = .011), miR‐148a‐3p (*p* = .032), and miR‐152‐3p (*p* = .013) were also observed in patients with stage I‐II, and miR‐130a‐5p (*p* = .002) and miR‐148a‐3p (*p* = .020) in patients with stage III‐IV (Figure S4). Stratified by T classification, we observed that significant difference in the expression of miR‐130a‐5p (*p* = .009), miR‐144‐3p (*p* = .042), miR‐148a‐3p (*p* = .029), and miR‐152‐3p (*p* = .024) between patients with clinical T1‐2 and the controls, as shown in Figure S5. The expression of miR‐23a‐3p level was significantly lower in BC with clinical T3‐4 than in the health controls (*p* = .003).

Expression of miR‐23a‐3p (*p* = .002), miR‐130a‐5p (*p* = .011), miR‐144‐3p (*p* = .002), miR‐148a‐3p (*p* = .008), and miR‐152‐3p (*p* = .008) was significantly different between patients without lymph node metastasis and the controls (Figure S6). MiR‐130a‐5p (*p* = .008) was also significantly different between patients with lymph node metastasis and the controls. Furthermore, miR‐148a‐3p expression was higher in patients with lymph node metastasis as compared to patients without lymph node metastasis (*p* = .030). Our results also suggested that patients with lymph node metastasis appeared to have higher expression of miR‐144‐3p (*p* = .041, Table 4).

In addition, lower levels of miR‐23a‐3p (*p* = .005) and miR‐152‐3p (*p* = .004) in patients with luminal A subtype, miR‐130a‐5p (*p* = .019) in patients with luminal B subtype and miR‐152‐3p (*p* = .010) in patients with Her‐2 subtype were observed compared with normal controls (Figure S7). MiR‐130a‐5p (*p* = .022) was also different between patients with Her‐2 subtype and patients with triple negative. The expression of miR‐152‐3p level was significantly lower in patients with luminal A subtype than in patients with luminal B subtype (*p* = .010).

### 3.6 miRNA expression and prognosis based on TCGA database

**Figure 3:**
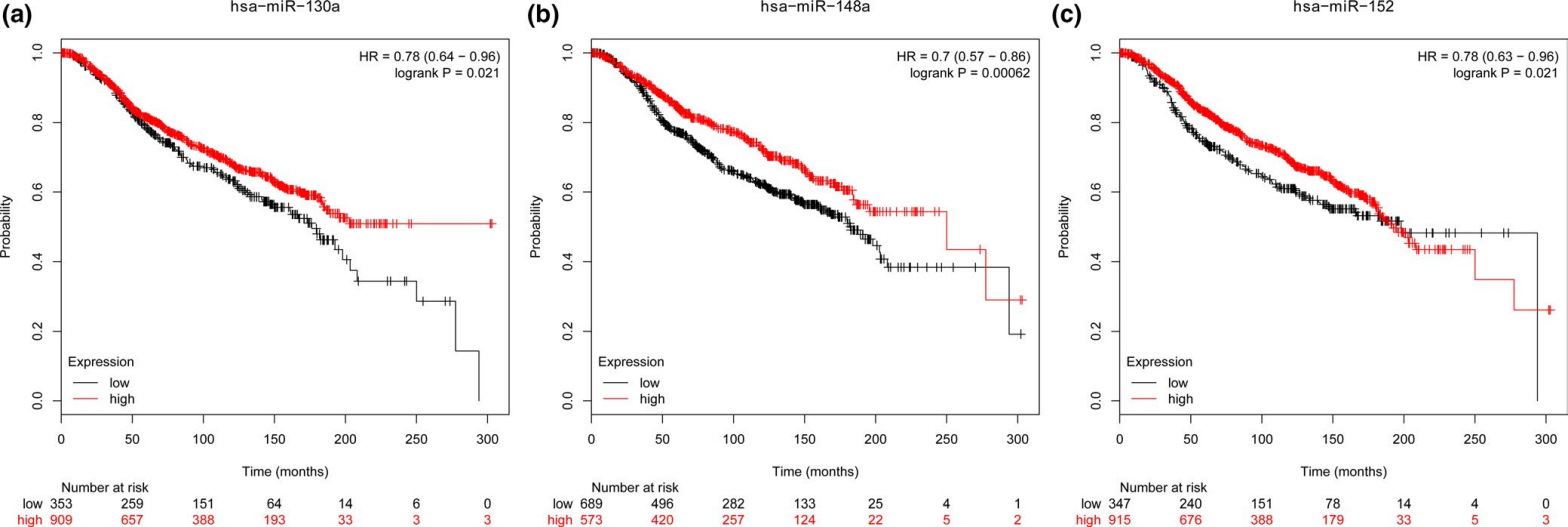
Kaplan–Meier Plotter of miRNA expression and breast cancer (BC) prognosis. The expression of miR‐130a‐5p (a), miR‐148a‐3p (b), and miR‐152‐3p (c) was associated with BC survival. The Kaplan–Meier plots were generated by Kaplan–Meier Plotter database (http://kmplot.com/analysis/index.php?p=service%26cancer=breast_mirna). *p*‐values (logrank test) .05 means the data is statistically significant

Based on Kaplan‐Meier Plotter database (Figure 3), the expression of miR‐130a‐5p [hazard ratio (HR) = 0.78; 95% CI: 0.64–0.96; logrank *p* = .021], miR‐148a‐3p (HR = 0.70; 95% CI: 0.57–0.86; logrank *p* = .00062), and miR‐152‐3p (HR = 0.78; 95% CI: 0.63–0.96; logrank *p* = .021) was found to be associated with BC prognosis.

## 4 DISCUSSION

An important area in current BC research is the identification of novel biomarkers for early diagnosis. Currently, several studies have demonstrated that circulating miRNAs are differentially expressed in patients with BC and are potential biomarkers useful in BC diagnosis (Grimaldi & Incoronato, 2019; Incoronato & Grimaldi, 2019; Swellam, El Magdoub, Hassan, Hefny, & Sobeih, 2018). In this study, we have identified significant reduction of miR‐23a‐3p, miR‐130a‐5p, miR‐144‐3p, miR‐148a‐3p, and miR‐152‐3p in the plasma of the patients compared with the controls, indicating the diagnostic potential of these miRNAs in BC.

In BC, miR‐23a was involved in invasion, migration, lymph node metastasis, and survival (Eissa, Matboli, & Shehata, 2015; Ma, Li, et al., 2017). Moreover, miR‐23a acted as negative regulators of PR expression in ER‐positive BC (Gilam et al., 2017). Several studies have suggested miR‐130a expression was downregulated in BC tissues and cells, and associated with the patients with recurrence (Sueta, Yamamoto, Mtt, Yamamoto‐Ibusuki, & Iwase, 2017). Besides, overexpression of miR‐130a was able to inhibit cell proliferation, invasion, and migration in MCF7 and MDA‐MB‐435 cells (Pan et al., 2015). MiR‐144 suppressed BC cell proliferation, migration, and invasion and induced cell cycle arrest and cell apoptosis by repressing CEP55 (Yin, Cai, Meng, Sui, & Jiang, 2018). Previous studies showed that miR‐148a could suppress the estrogen‐induced viability and migration of BC cells via inhibition of ERα protein expression (Ma, Feng, et al., 2017). MiR‐152‐3p might serve as a tumor suppressor in human BC cells via negatively regulating PIK3CA expression to inhibit the activation of AKT and RPS6, leading to suppression of BC cell proliferation (Ge, Wang, Kong, Gao, & Sun, 2017). These studies have indicated that miR‐23a‐3p, miR‐130a‐5p, miR‐144‐3p, miR‐148a‐3p, and miR‐152‐3p played important roles in human BC tumorigenesis.

Plasma levels of miRNA are not necessarily a reflection of tissue levels (Cookson et al., 2012). We also compared the differences in miRNA expression levels in tumor tissue versus plasma. Notably, miR‐130a‐5p, miR‐144‐3p, and miR‐152‐3p were downexpressed in BC tissues as well as plasma; therefore, these miRNAs might directly reflect BC tumor biology. We also found that the relative expression of these five miRNAs was closely associated with the clinicopathologic features of the BC such as the expression of sex hormone receptor (ER, PR, and HER2), histological tumor grades, and lymph node metastasis. In clinic, the expression level of hormone receptors including ER, PR, and HER2 and tumor grade is often used for classification and target therapy indicators of BCs (Schettini et al., 2016). Our results showed that the expression of miR‐23a‐3p, miR‐144‐3p, and miR‐152‐3p was related to ER positive and PR positive, indicating that these plasma miRNA levels might separate the ER‐positive and PR‐positive subtypes of BC. Stratified by BC stage, miR‐23a‐3p, miR‐144‐3p, and miR‐152‐3p did show significant differences in the staging compromised to the control, especially in stage I‐II. Besides, the expression of the miR‐130a‐5p, miR‐144‐3p, miR‐148a‐3p, and miR‐152‐3p in the plasma showed a significant difference between patients with T1‐2 and the controls. Moreover, we also found that miR‐144‐3p and miR‐148a‐3p were associated with lymph node invasion. Consistent with our results, one study indicated that miR‐144‐3p served as early detection markers of metastasis in BC (Madhavan et al., 2016). The findings suggest that the deregulation of the miRNAs might affect critical molecular events involved in tumor progression and could be used to identify the different nature of breast tissues. Thus, measurements of miRNAs as the biochemical markers can help to diagnose the different stages of BC prior to clinical investigations on the samples. However, further studies in a large population should be needed to validate the feasibility of these miRNAs as novel noninvasive biomarkers.

Our study presents several limitations. First, the size of patients with BC and normal controls were still relatively small, limiting the evaluation on these miRNAs as predictive biomarkers in early detection of BC. Second, the follow‐up results, such as disease recurrence and disease‐free survival were lacked, which prevented us from conducting prognosis analyses. Further validations in large, independent prospective cohorts are recommended.

## 5 CONCLUSION

In conclusion, we identified five miRNAs as being downregulated in the plasma of patients with BC compared to healthy control individuals. Strikingly, expression trends for miR‐130a‐5p, miR‐144‐3p, and miR‐152‐3p were similar in both tissue and plasma. Besides, they were also significantly associated with sex hormone receptor, clinical stage, and lymph node metastasis. All of plasma miRNAs them exhibit great potential in serving as promising noninvasive candidates for BC detection. Future prospective studies using a large cohort will be needed to confirm our findings.

## CONFLICT OF INTEREST

The authors declare that there are no competing interests regarding the publication of this paper.

## AUTHORS CONTRIBUTION

The work presented here was carried out in collaboration between all authors. Xu Li and Wenjing Zou carried out the molecular genetic studies and drafted the manuscript. Yuzhen Wang and Zijun Liao performed the statistical analyses and interpreted the results. Lina Li, Yang Zhai, and Lingxiao Zhang collected clinical information about patients and performed the extraction of plasma RNA and miRNA qRT‐PCR experiments. Shanzhi Gu and Xinhan Zhao conceived the study, worked on associated data collection and their interpretation, participated in the design and coordination of the study, and funded the study. All authors read and approved the final manuscript.
