# Supplementary figures and images for: Plasma‐based microRNA signatures in early diagnosis of breast cancer
Source: Mol Genet Genomic Med. 2020 Mar 2;8(5):e1092. doi: 10.1002/mgg3.1092 (PMC7216817; doi:10.1002/mgg3.1092)

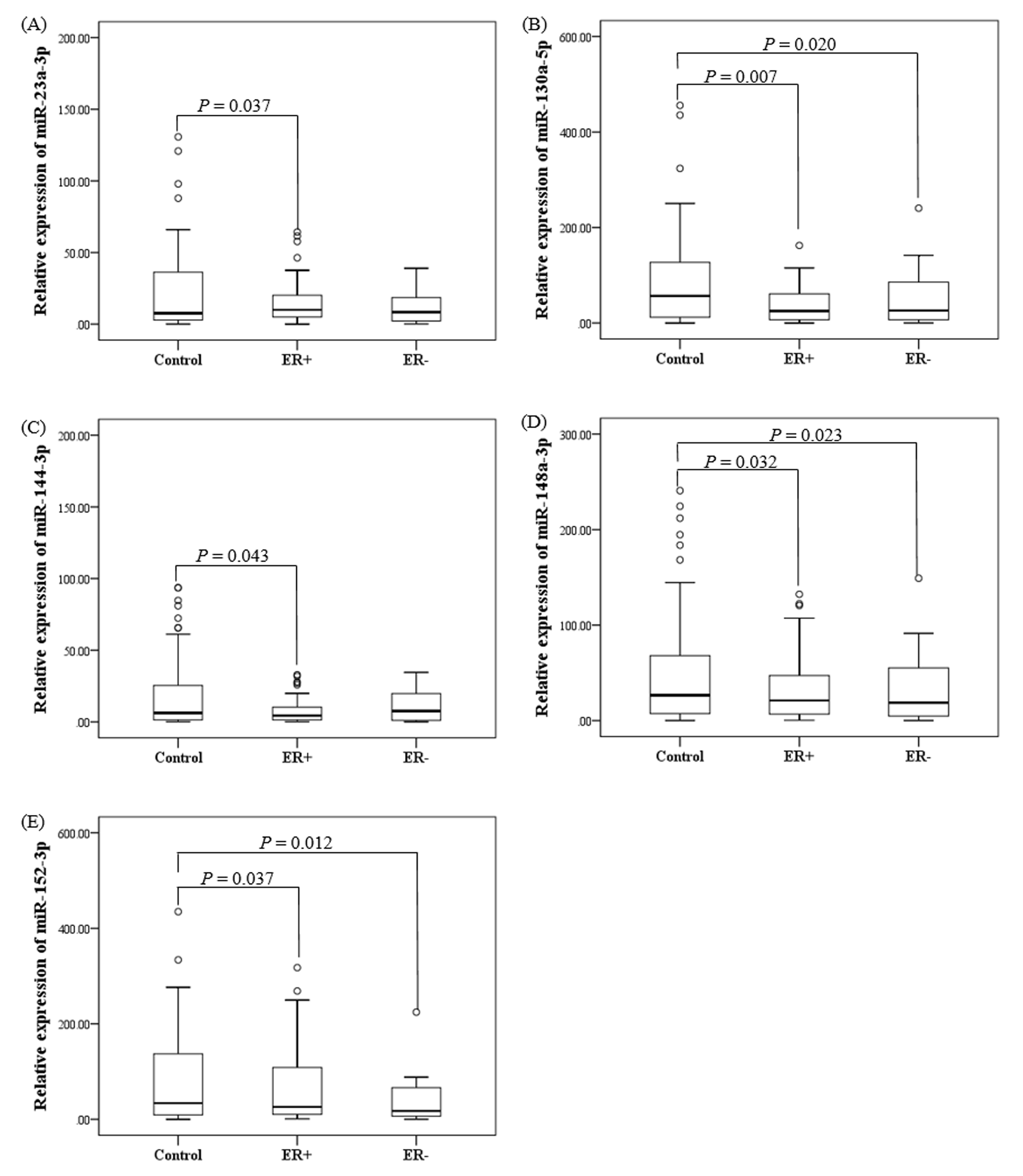

Supplement: Supplementary file 1 [file MGG3-8-e1092-s001.tif]

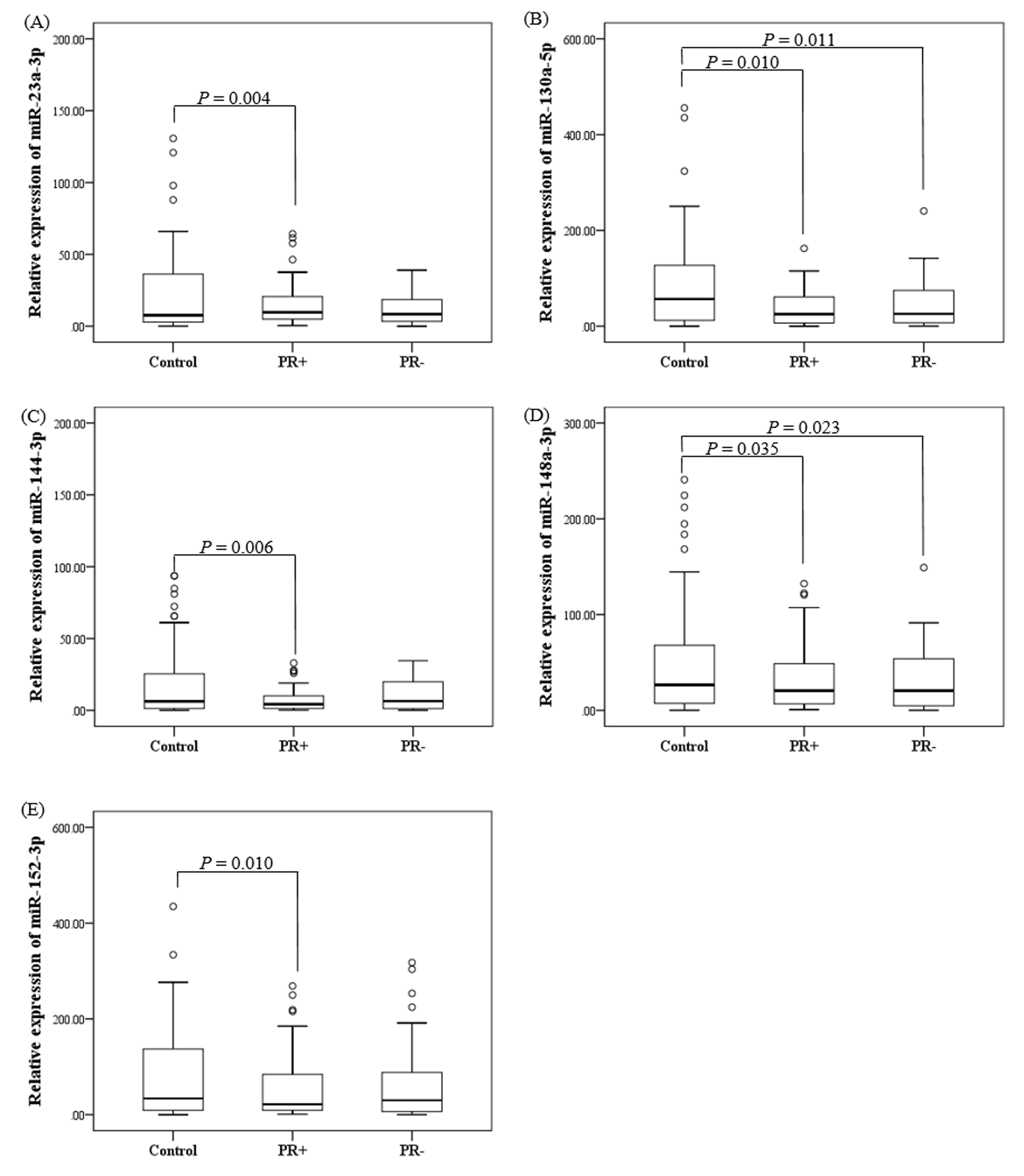

Supplement: Supplementary file 2 [file MGG3-8-e1092-s002.tif]

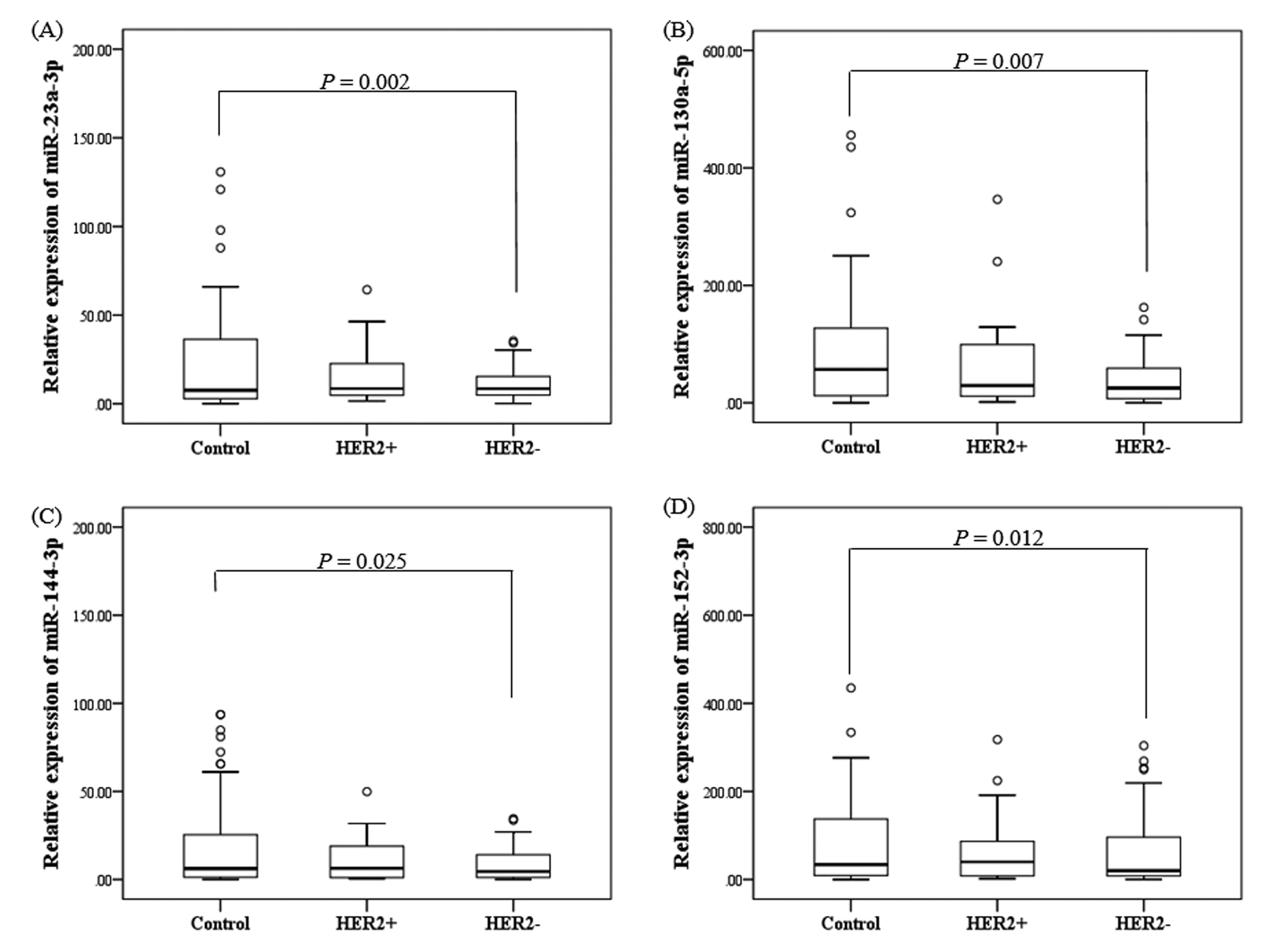

Supplement: Supplementary file 3 [file MGG3-8-e1092-s003.tif]

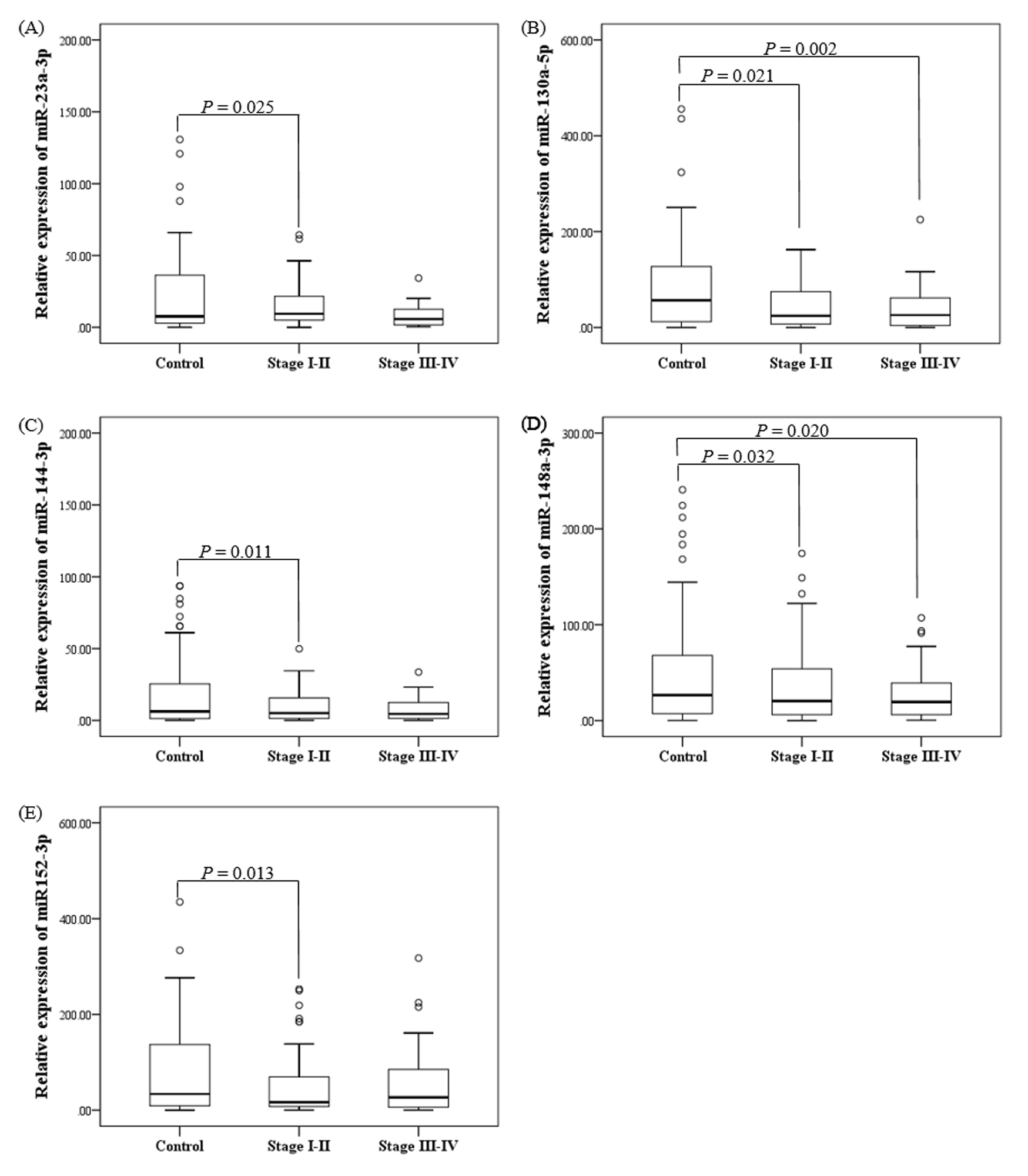

Supplement: Supplementary file 4 [file MGG3-8-e1092-s004.tif]

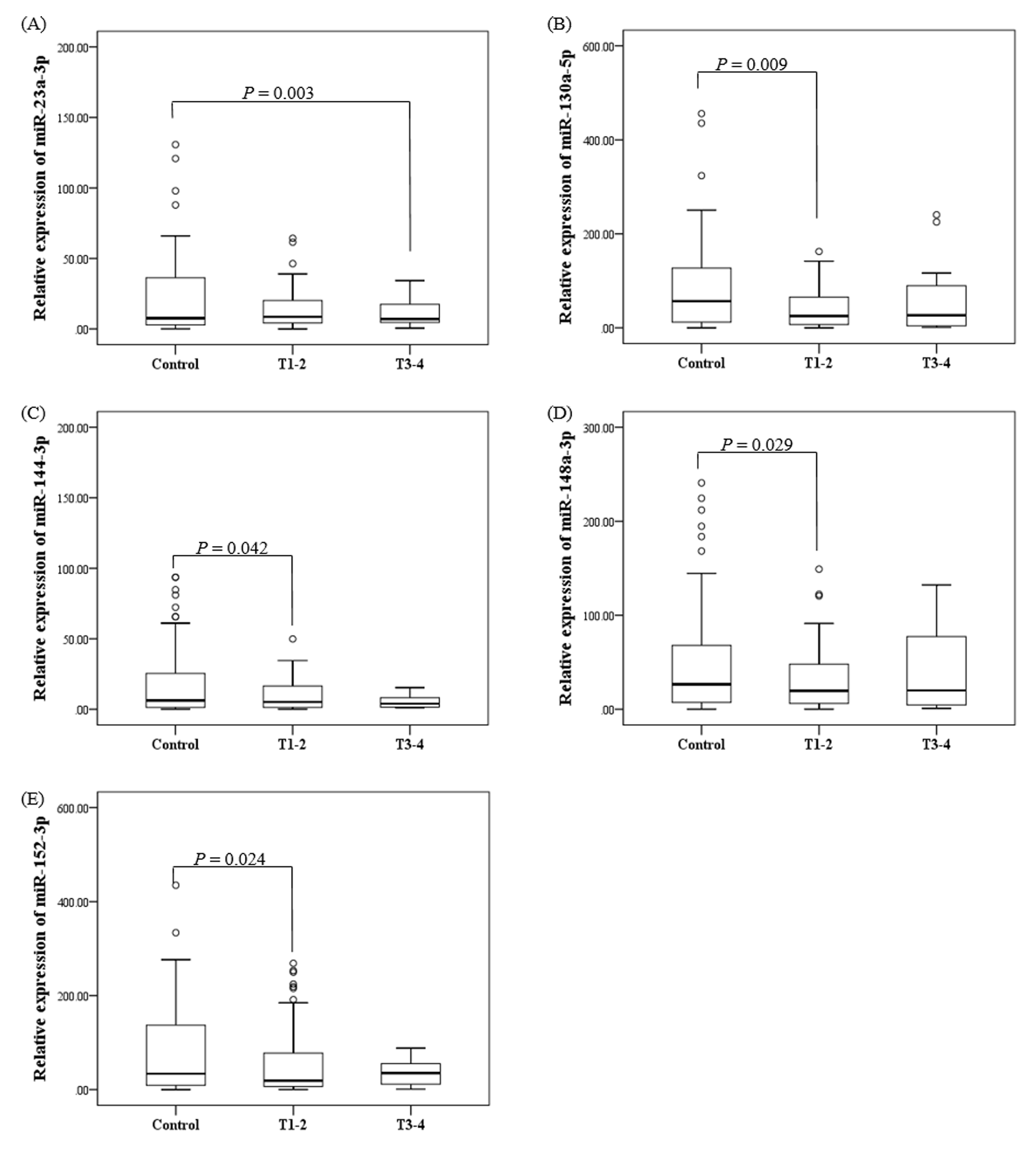

Supplement: Supplementary file 5 [file MGG3-8-e1092-s005.tif]

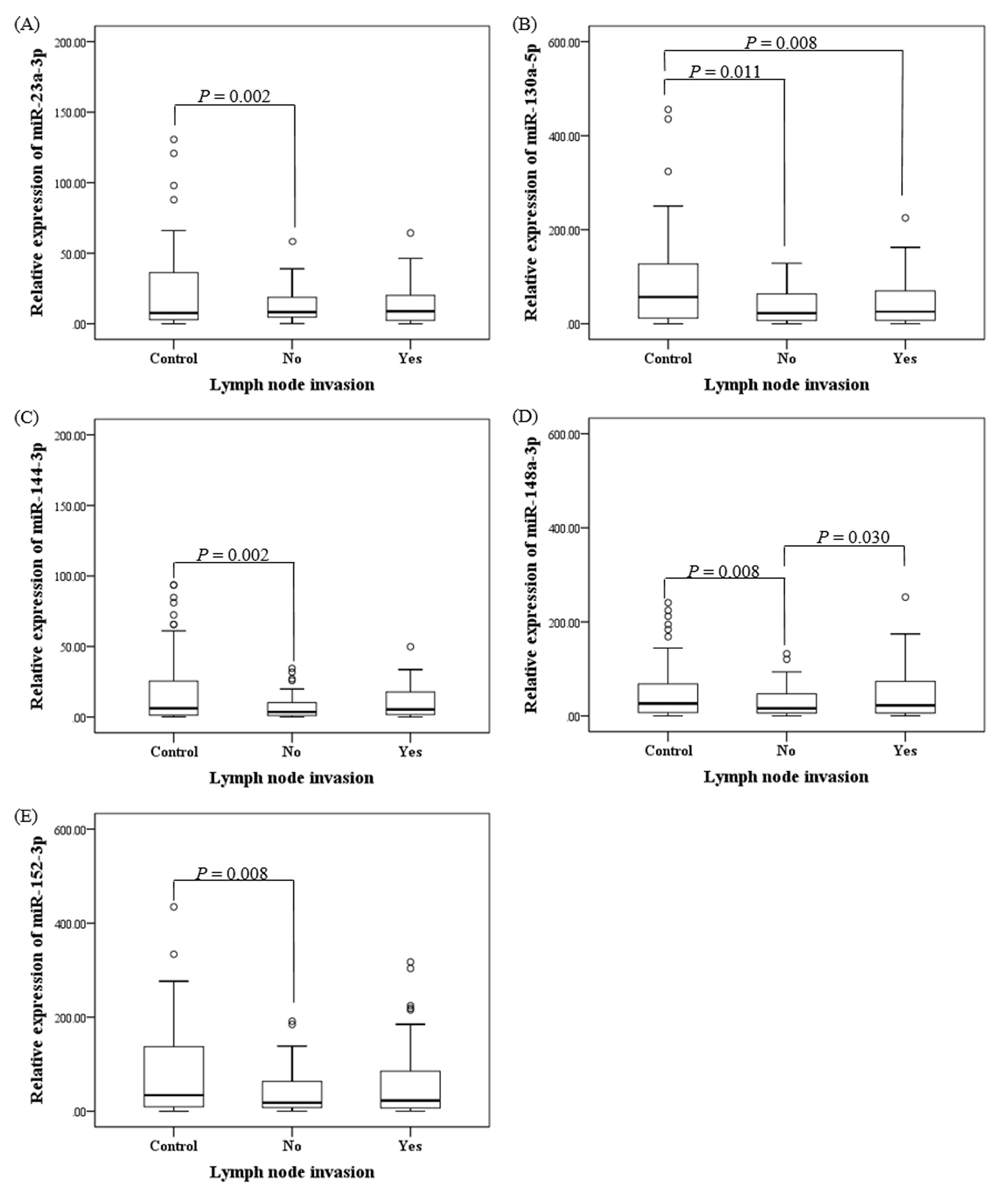

Supplement: Supplementary file 6 [file MGG3-8-e1092-s006.tif]

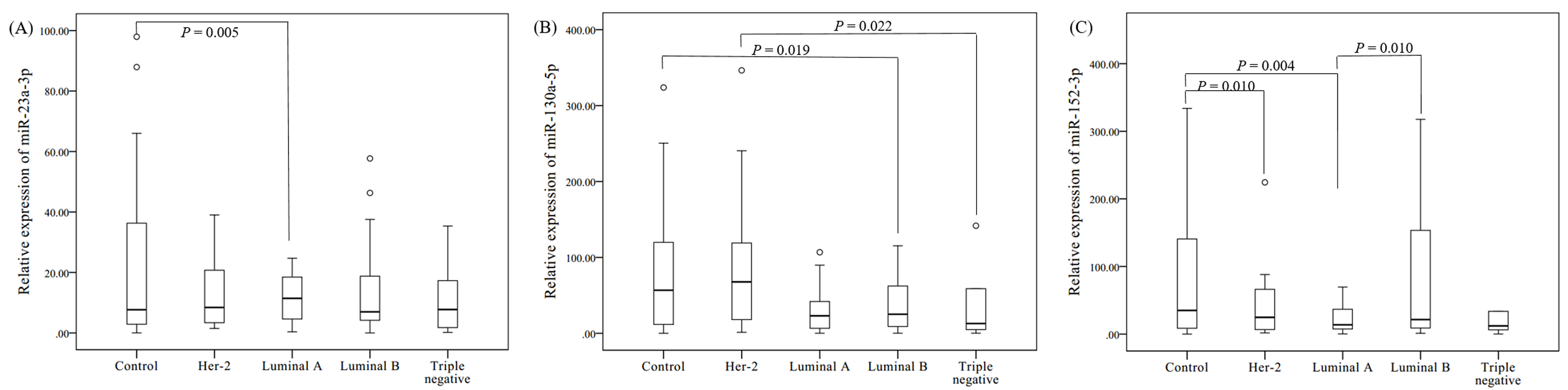

Supplement: Supplementary file 7 [file MGG3-8-e1092-s007.tif]
