# Supplementary material for: Plasma‐based microRNA signatures in early diagnosis of breast cancer
Source: Mol Genet Genomic Med. 2020 Mar 2;8(5):e1092. doi: 10.1002/mgg3.1092 (PMC7216817; doi:10.1002/mgg3.1092)
Supplement: Supplementary file 8 [file MGG3-8-e1092-s008.docx]

**Table S1. Forward Primers for quantitative RT-PCR.**

| **miRNAs** | **Forward primer** |
| --- | --- |
| miR-23a-3p | ccgATCACATTGCCAGGGATTT |
| miR-29b-2-5p | agCTGGTTTCACATGGTGGCTTAG |
| miR-130a-5p | cgcGCTCTTTTCACATTGTGCTACT |
| miR-144-3p | ccgcgcgTACAGTATAGATGATGTACT |
| miR-148a-3p | cgcgTCAGTGCACTACAGAACTTTGT |
| miR-152-3p | ccgTCAGTGCATGACAGAACTTGG |
| miR-182-5p | cgTTTGGCAATGGTAGAACTCACACT |
